# Postoperative nosocomial infections among children with congenital heart disease

**DOI:** 10.12669/pjms.303.4648

**Published:** 2014

**Authors:** Jian Zhang, Yan Yuan, Peiling Li, Tuanjie Wang, Jun Gao, Jinhua Yao, Shujun Li

**Affiliations:** 1 Jian Zhang, Pediatric Intensive Care Unit, The First Affiliated Hospital of Xinxiang Medical University Weihui 453100, P. R. China.; 2 Yan Yuan, Department of Neurology, The First Affiliated Hospital of Xinxiang Medical University Weihui 453100, P. R. China.; 3 Peiling Li, Department of Pediatrics, The First Affiliated Hospital of Xinxiang Medical University Weihui 453100, P. R. China.; 4 Tuanjie Wang, Pediatric Intensive Care Unit, The First Affiliated Hospital of Xinxiang Medical University Weihui 453100, P. R. China.; 5 Jun Gao, Neonatal Intensive Care Unit, The First Affiliated Hospital of Xinxiang Medical University Weihui 453100, P. R. China.; 6 Jinhua Yao, Pediatric Intensive Care Unit, The First Affiliated Hospital of Xinxiang Medical University Weihui 453100, P. R. China.; 7 Shujun Li, Pediatric Intensive Care Unit, The First Affiliated Hospital of Xinxiang Medical University Weihui 453100, P. R. China.

**Keywords:** Congenital heart disease, Nosocomial infection, Risk factor

## Abstract

***Objective:*** To study the pathogen distribution, antimicrobial susceptibility and risk factors of postoperative nosocomial infections among children with congenital heart disease.

***Methods:*** Three hundreds children with congenital heart disease admitted to our hospital to receive surgeries from February 2010 to February 2013 were selected.

***Results:*** A total of 120 children were tested as positive by sputum culture, with the infection rate of 40.0%. The top five most common pathogenic microorganisms included *Staphylococcus epidermidis*, *Staphylococcus aureus*, *Enterococcus*, *Pseudomonas aeruginosa*, and *Candida albicans*. *S. epidermidis*, *S. aureus* and *Enterococcus* were highly resistant to penicillin, azithromycin and erythromycin, moderately susceptible to levofloxacin and cefazolin, and completely susceptible to vancomycin. Multivariate Logistic regression analysis showed that hospitalization stay length, combined use of antibiotics, systemic use of hormones, mechanical ventilation and catheter indwelling were the independent risk factors of postoperative nosocomial infections (P<0.05).

***Conclusion: ***Nosocomial infection, which was the most frequent postoperative complication of pediatric congenital heart disease, was predominantly induced by Gram-positive bacteria that were highly susceptible to cephalosporins and vancomycin. Particular attention should be paid to decrease relevant risk factors to improve the prognosis.

## INTRODUCTION

Congenital heart disease (CHD), which results from embryonic cardiovascular abnormalities, threatens about 0.7%-0.8% of Chinese children.^1^^-^^3^ With disease spectrum changes, birth defects have become the leading causes of perinatal deaths in China, of which CHD accounts for 1/3 as the most common pediatric heart disease.^4^ CHD greatly burdens society and families due to critical conditions, numerous complications, tricky treatment and high mortality rate.^5^ Meanwhile, CHD children, who suffer from left-right shunt, are prone to being complicated with recurrent respiratory infections owing to increased blood flow in pulmonary circulation. Besides, CHD children were vulnerable to nosocomial infections because of developmental retardation, low immunity, long-term use of antibiotics and ventilator-assisted breathing, etc.^6^^,^^7^ Hence, prognosis is seriously affected with elevated mortality rate due to considerable medical expenses and suffering.^8^^,^^9^ Therefore, we herein aimed to investigate the pathogen distribution, antimicrobial susceptibility and risk factors of postoperative nosocomial infections among the CHD children admitted to our hospital.

## METHODS

***General Information: ***All the experiments was approved by the ethic committee of The First Affiliated Hospital of Xinxiang Medical University, and consent has been obtained from all patients. Three hundreds of CHD children admitted to our hospital to receive surgeries from February 2010 to February 2013 were selected. ***Inclusion criteria:*** Diagnosed clinically by color Doppler echocardiography, X-ray examination and surgery; long-term residents; younger than 10 years old; consent has been obtained from guardians. ***Exclusion criteria:*** Pulmonary infection one week before surgery; abnormalities in positions other than the cardiovascular system. The patients included 154 boys and 146 girls, aged from 3 to 9 years old [average: (6.56±0.33) years old]. ***Onset types:*** 102 cases of ventricular septal defect, 98 cases of atrial septal defect, 30 cases of pulmonary hypertension and stenosis, 20 cases of patent ductus arteriosus, 20 cases of tetralogy of Fallot, and another 30 cases (including 16 cases of transposition of the great arteries, 10 cases of coarctation of the aorta and 4 cases of anomalous pulmonary venous drainage).

***Microbial culture: ***All children received surgeries, the sputa of whom were gently collected by a chopstick wrapped with a small piece of gauze on the top. Then the sputa were cultured by BacT/Albert 120 microbial culture system (Netherlands). The samples were inoculated onto chocolate agar plates by streaking and cultured for 24-48 h. Possible pathogenic microorganisms were further identified.

***Antimicrobial susceptibility analysis: ***Antimicrobial susceptibility was identified by an API analyzer (BioMérieux, France) based on the most updated National Committee for Clinical Laboratory Standards (NCCLS).

***Investigation contents: ***The children and their parents were investigated by questionnaires to find out the possible risk factors, with the incomplete ones being excluded. The investigation contents included: 1) General information of children: Name, gender, date of birth, birth weight, ethnic group, clinical diagnosis, hospitalization stay length, use of antibiotics, use of hormones; intravenous nutritional intervention; mechanical ventilation and catheter indwelling; 2) Early pregnancy period of mothers: History of diseases, medication history, history of exposure to harmful substances, adverse psychosocial factors and history of threatened abortion; 3) Living style of mothers: Smoking, second-hand smoking, drinking, drug addiction, and uses of computer and cell phone; 4) Reproductive history of mothers: Reproductive age and adverse reproductive history; 5) Living style of fathers: Smoking, second-hand smoking, drinking, drug addiction, and uses of computer and cell phone; 6) Family history: CHD history of third-degree relatives, and history of consanguineous marriage; 7) Living environment: Air and water pollutions. The effective rate of this investigation was 100.0%.

***Statistical analysis: ***All data were analyzed by SAS13.0. The investigation contents were first subjected to univariate analysis, and the significant factors were further subjected to multivariate Logistic regression analysis. P<0.05 was considered statistically significant.

## RESULTS

***General infection status: ***A total of 120 children were tested as positive by sputum culture, with the infection rate of 40.0%. Of the 150 isolated pathogenic microbial strains, there were 100 Gram-positive bacteria, 40 Gram-negative bacteria and 10 fungi. The top 5 most common pathogenic microorganisms included *Staphylococcus epidermidis*, *Staphylococcus aureus*, *Enterococcus*, *Pseudomonas aeruginosa*, and *Candida albicans* (Table-I).

***Antimicrobial susceptibility analysis: ***The susceptibilities of *S. epidermidis*, *S. aureus* and *Enterococcus* against penicillin, azithromycin, erythromycin, levofloxacin, cefazolin and vancomycin were analyzed. The three strains were highly resistant to penicillin, azithromycin and erythromycin, moderately susceptible to levofloxacin and cefazolin, and completely susceptible to vancomycin (Table-II).

***Risk factors of nosocomial infections: ***Multivariate Logistic regression analysis suggested that hospitalization stay length, combined use of antibiotics, systemic use of hormones, mechanical ventilation and catheter indwelling were the independent risk factors of postoperative nosocomial infections (P<0.05) (Table-III).

## DISCUSSION

Recently, significantly more neonates survive owing to the advances in medical technology. However, the incidence rate of CHD accounts for approximately 7%, of which ventricular septal defect, atrial septal defect, patent ductus arteriosus and tetralogy of Fallot are the main causes.^10^ In this study, there were 102 cases of ventricular septal defect, 98 cases of atrial septal defect, 30 cases of pulmonary hypertension and stenosis, 20 cases of patent ductus arteriosus, 20 cases of tetralogy of Fallot, and another 30 cases.

**Table-I:** Pathogen distribution of postoperative nosocomial infections

*Pathogen*	*Strain number (n=150)*	*Proportion*
S. epidermidis	48	32.0%
S. aureus	32	21.3%
Enterococcus	18	12.0%
P. aeruginosa	12	8.0%
C. albicans	8	5.3%
Others	42	28.0%

**Table-II:** Antimicrobial susceptibility analysis (n).

*Antibiotics*	*S. epidermidis (n=48)*			*S. aureus (n=32)*			*Enterococcus (n=18)*		
	*Resistance*	*Susceptibility*	*Resistance rate*	*Resistance*	*Susceptibility*	*Resistance rate*	*Resistance*	*Susceptibility*	*Resistance rate*
Penicillin	48	0	100.0%	32	0	100.0%	18	0	100.0%
Azithromycin	44	4	91.7%	26	6	81.3%	18	0	100.0%
Erythromycin	48	0	100.0%	25	7	78.1%	18	0	100.0%
Levofloxacin	28	20	58.3%	24	8	75.0%	9	9	50.0%
Cefazolin	6	42	12.5%	20	12	62.5%	9	9	50.0%
Vancomycin	0	48	0.0%	0	32	0.0%	0	18	0.0%

**Table-III:** Independent risk factors of nosocomial infections

*Factor*	*P value*	*Odds ratio (OR)*	*95% confidential interval*
Hospitalization stay length	0.004	6.263	1.786-21.863
Combined use of antibiotics	0.003	5.008	1.750-14.652
Systemic use of hormones	0.000	3.236	1.145-6.521
Mechanical ventilation	0.001	1.582	1.132-3.069
Catheter indwelling	0.008	6.251	0.952-1.559

As evidenced by the Human Genome Project, the interaction between genetic susceptibility and environmental teratogens mainly contributes to CHD. Moreover, there may be new risk factors emerging with altered environment in this region. For instance, long-term uses of computer and cell phone, discharge of industrial contaminants, as well as water and air pollutions may also lead to CHD, but whether they are the risk factors remain unraveled.^11^^,^^12^

 Pediatric cardiac surgery gives rise to evidently reduced systemic immunity, increased risks of postoperative infections and unsatisfactory prognosis by leaving major traumas. Identifying pathogens by sputum culture can raise the successful rate of surgery by early administration of effective antibiotics.

CHD complicated with lower respiratory infections may prevent surgeries from being successful.In the meantime, CHD may be diagnosed as pneumonia by mistake owing to the subtle early signs, thus not benefiting the postoperative recovery.^13^ A total of 120 children were tested as positive by sputum culture, with the infection rate of 40.0%. Of the 150 isolated pathogenic microbial strains, there were 100 Gram-positive bacteria, 40 Gram-negative bacteria and 10 fungi. The top 5 most common pathogenic microorganisms included *Staphylococcus epidermidis*, *Staphylococcus aureus*, *Enterococcus*, *Pseudomonas aeruginosa*, and *Candida albicans*. The detection rates of Gram-positive bacteria exceeded those of Gram-negative ones, which may be associated with thymic hypoplasia, low immunity, and fragile barrier function, etc. *S. epidermidis*, an opportunistic pathogen, hides in sebaceous glands, sweat glands and skin folds under normal conditions and invades blood after puncture, leading to nosocomial infections eventually.^14^ *S. aureus* results in severe complications such as endocarditis, osteomyelitis and arthritis, as well as brings about dysbiosis by inhibiting normal microorganisms in human body. Gram-positive bacteria were highly resistant to penicillin, azithromycin and erythromycin, moderately susceptible to levofloxacin and cefazolin, and completely susceptible to vancomycin. Generally, all Gram-positive bacteria are resistant to macrolides and penicillin (over 90%), and highly and entirely susceptible to cefazolin and vancomycin respectively.^15^ At present, vancomycin is still given first priority in treating the infections of methicillin-resistant *S. aureus*, coagulase-negative staphylococci and other enterococci.

Multivariate Logistic regression analysis revealed that length of hospitalization, combined use of antibiotics, systemic use of hormones, mechanical ventilation and catheter indwelling were the independent risk factors of postoperative nosocomial infections (P<0.05). Currently, irrational use of antibiotics rapidly increases opportunistic pathogens and aggravates antibiotic resistance. Meanwhile, combined administration of antibiotics gives rise to dysbiosis by killing or suppressing normal microorganisms other than target bacteria. Glucocorticoids can inhibit the functions of polymorphonuclear leukocytes, mononuclear macrophages and T cells, thus inducing nosocomial infections. Moreover, mechanical ventilation for more than two days subjects patients to fungal colonization of the respiratory tract. Furthermore, inserting straight catheters, which involves invasive operations, is also related with nosocomial infections.^16^

In summary, Gram-positive bacteria, which were the dominant species contributing to nosocomial infection as the most frequent complication of CHD, were highly resistant to cephalosporins and vancomycin. Hence, it is crucial to minimize the associated risk factors to optimize the surgical outcomes.

## Authors’ contributions:

**JZ and SJL:** Study design and manuscript preparation.

**YY, PLL, TW, JG and JHY:** Data collection and analysis.
